# HbA1c of 5.8% or higher as the most useful indicator for recommendation of ultrasonography to detect nonalcoholic fatty liver disease

**DOI:** 10.1002/jgh3.13019

**Published:** 2023-12-11

**Authors:** Miwa Tatsuta, Masafumi Ono, Shungo Kimura, Kaori Zuigyo, Yudai Sato, Akemi Tomida, Mitsuyoshi Kobayashi, Ritsuko Yoshikawa, Satoshi Murao, Joji Tani, Asahiro Morishita, Hideki Kobara, Takashi Himoto, Tsuyoshi Maeta, Yoshihiro Mori, Fumikazu Kohi, Tsutomu Masaki

**Affiliations:** ^1^ Department of Gastroenterology KKR Takamatsu Hospital Kagawa Japan; ^2^ Department of Gastroenterology and Neurology Faculty of Medicine, Kagawa University Kagawa Japan; ^3^ Division of Innovative Medicine for Hepatobiliary & Pancreatology Faculty of Medicine, Kagawa University Kagawa Japan; ^4^ Department of Endocrinology KKR Takamatsu Hospital Kagawa Japan; ^5^ Department of Medical Technology Kagawa Prefectural University of Health Sciences Kagawa Japan; ^6^ Department of Internal Medicine KKR Takamatsu Hospital Kagawa Japan

**Keywords:** diabetes mellitus, HbA1c, nonalcoholic fatty liver disease, ultrasonography

## Abstract

**Background and Aim:**

Nonalcoholic fatty liver disease (NAFLD) is closely associated with metabolic syndrome. This study was performed to examine the association between NAFLD and each factor of metabolic syndrome and to identify the factors that are most strongly associated with NAFLD in participants undergoing health checkups.

**Methods:**

We studied 6538 participants who underwent a health checkup from 2017 to 2018 in our institution. Participants with alcohol intake exceeding 20 g/day or with other chronic liver diseases were excluded. Fatty liver was detected by ultrasonography.

**Results:**

In total, 4310 participants were enrolled, and 28.4% had fatty liver (NAFLD). The prevalence of NAFLD was highest in the diabetes mellitus (DM)‐only group than in the dyslipidemia‐only or hypertension‐only group. The DM‐only group was the only group whose prevalence of NAFLD was >50% in the overall study and in males. The prevalence of NAFLD was higher in males than in females in the DM‐only, hypertension‐only, and dyslipidemia‐only groups. The prevalence of NAFLD was >70% in the dyslipidemia and DM combined group. Multivariate analysis showed that gender and HbA1c were the independent factors most strongly associated with NAFLD. The cutoff value for HbA1c by receiver operating characteristic curve analysis was 5.8% (sensitivity, 57.9%; specificity, 72.6%; area under the curve, 0.70).

**Conclusion:**

NAFLD was most strongly associated with DM, among the various components of metabolic syndrome. We strongly recommend abdominal ultrasonography to detect NAFLD in patients with an HbA1c of ≥5.8% in general practice and during health checkups.

## Graphical abstract

This study was performed to examine the association between NAFLD and each factor of metabolic syndrome and to identify the factors that are most strongly associated with NAFLD in participants undergoing health checkups. NAFLD was most strongly associated with DM among the various components of metabolic syndrome. We strongly recommend abdominal ultrasonography to detect NAFLD in patients with an HbA1c of ≥5.8% in general practice and during health checkups.

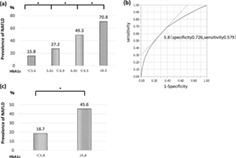

## Introduction

Nonalcoholic fatty liver disease (NAFLD) is mainly associated with factors related to metabolic syndrome and is characterized by the presence of fatty liver on histology exam or imaging. NAFLD is classified into nonalcoholic fatty liver (NAFL) and nonalcoholic steatohepatitis (NASH), which have recently been found to be mutually transitioning. The most prognostically relevant pathological finding is liver fibrosis,^1^ and the follow‐up and treatment options depend on the degree of fibrosis.

Cardiovascular disease, but not liver disease‐related conditions, is the most common cause of death in patients with NAFLD.^1^ A follow‐up study of the prognosis of 10 000 patients who underwent liver biopsy showed that the NAFLD group had a 1.7‐fold higher risk of death than the control group.^2^ The worldwide prevalence of NAFLD is 25%, and NASH may account for 20–25% of these cases of NAFLD.^3^ The prevalence of NAFLD among participants undergoing health checkups in Japan is 29.7%.^4^ An estimated 20 million people suffer from NAFLD in Japan. The number of patients with NAFLD is increasing, and a recent paper estimated that by 2030, there will be 23 million people with NAFLD in Japan and more than 1 million people with advanced fibrosis (stage F3 and F4 NAFLD).^5^

The main background factors of NAFLD/NASH are insulin resistance, metabolic syndrome, and related diseases such as type 2 diabetes mellitus (DM), dyslipidemia, and hypertension.^6^ Metabolic syndrome is a risk factor for NAFLD, and a prospective cohort study of 4401 patients who underwent health screening showed that NAFLD progression was 4.0‐fold and 11.2‐fold higher in males and females with metabolic syndrome, respectively, than in those without metabolic syndrome.^7^ Conversely, NAFLD is an independent risk factor for each of the factors of metabolic syndrome (obesity, DM, dyslipidemia, and hypertension).^8^ In one study, 88% of patients with NASH and 53% of those with NAFL had metabolic syndrome,^9^ and most patients with NAFLD have a background of metabolic syndrome factors. The present study was performed to examine the association between each factor of metabolic syndrome and NAFLD in participants undergoing health checkups at a single institution and to identify the factors that are most strongly associated with NAFLD.

## Patients and methods

### Study population

We studied 6538 individuals (61.3% male) aged 20–87 years (mean, 51.0 years) who received a health checkup in KKR Takamatsu Hospital. Individuals were included if they fulfilled the following criteria: (i) they had undergone abdominal ultrasonography; (ii) they did not have alcoholic liver disease (alcohol intake of ≥20 g/day); (iii) they had no markers of hepatitis B virus infection (hepatitis B surface antigen) or hepatitis C virus infection (anti‐hepatitis C virus antibodies); and (iv) they had no history of hepatitis B virus infection, hepatitis C virus infection, autoimmune hepatitis, or primary biliary cholangitis. Finally, 4310 individuals who met the inclusion criteria were enrolled. This single‐center, retrospective, observational study was conducted from April 2017 to March 2018. All procedures followed were in accordance with the Helsinki Declaration of 1975, as revised in 2008. Informed consent was obtained from all patients for being included in the study. The study was approved by the Clinical Ethics Committee of KKR Takamatsu Hospital (Registration No. E226).

### Diagnostic criteria for dyslipidemia, hypertension, and DM

Dyslipidemia was diagnosed based on a low‐density lipoprotein cholesterol (LDL‐C) concentration of ≥140 mg/dL, high‐density lipoprotein cholesterol (HDL‐C) concentration of <40 mg/dL, triglyceride concentration of ≥150 mg/dL, or current treatment.^10^ Hypertension was diagnosed based on a systolic blood pressure of ≥140 mmHg, diastolic blood pressure of ≥90 mmHg, or current treatment.^11^ DM was diagnosed based on a fasting blood glucose concentration of ≥126 mg/dL, HbA1c of ≥6.5%, or current treatment.^12^

### Definition of fatty liver by abdominal ultrasonography

Bright liver was defined as 0–2 points. The diagnosis of bright liver was based on abnormally intense, high‐level echoes arising from the hepatic parenchyma and was graded as none, mild, or severe in accordance with the intensity. Hepatorenal echo contrast was defined as 0 to 1 point. The diagnosis of hepatorenal echo contrast was based on evident ultrasonographic contrast between the hepatic and right renal parenchyma on the right intercostal sonogram at the midaxillary line. Deep echo attenuation was defined as 0–2 points. The diagnosis of deep echo attenuation was based on evident attenuation of echo penetration into the deep portion of the liver and impaired visualization of the diaphragm. Vessel blurring was defined as 0–2 points. The diagnosis of vessel blurring was based on impaired visualization of the borders of intrahepatic vessels and narrowing of their lumen. Blurring of the boundary between the liver and the right kidney or gallbladder wall was defined as 0–2 points. A total of ≥2 points was considered to be diagnostic for fatty liver.^13^ Ultrasonography was performed with the following units: Xario 200 with a 6‐MHz convex array transducer (Canon Medical Systems, Otawara, Japan) and Aplio XG with a 6‐MHz convex array transducer (Canon Medical Systems).

### Statistical analysis

The prevalence of NAFLD among participants with dyslipidemia, hypertension, and DM was determined, and the *P*‐value between each group was calculated using the Chi‐squared test. A *P*‐value of <0.005 was considered statistically significant. The characteristics of non‐fatty liver and NAFLD were analyzed using the Wilcoxon signed‐rank test, and a *P*‐value of <0.0001 was considered statistically significant. To identify NAFLD‐related factors, multivariate analyses were performed for 16 items: age, gender, body mass index (BMI), body fat percentage, waist circumference, systolic blood pressure, diastolic blood pressure, aspartate aminotransferase (AST), alanine aminotransferase (ALT), LDL‐C, HDL‐C, triglycerides, fasting glucose, fasting insulin, homeostatic model assessment for insulin resistance (HOMA‐IR), and HbA1c. Logistic regression analysis was used, and a *P*‐value of <0.005 was considered statistically significant. The prevalence of NAFLD among participants with different HbA1c levels was examined, and the *P*‐values between groups were determined using the Chi‐squared test. A *P*‐value of <0.0001 was considered statistically significant. The cut‐off value for HbA1c was determined by receiver operating characteristic (ROC) analysis. The optimal cut‐off value was calculated using Youden's index. We used JMP11 software to perform the statistical analysis.

## Results

### Prevalence of NAFLD among participants undergoing health checkups

**Figure 1:**
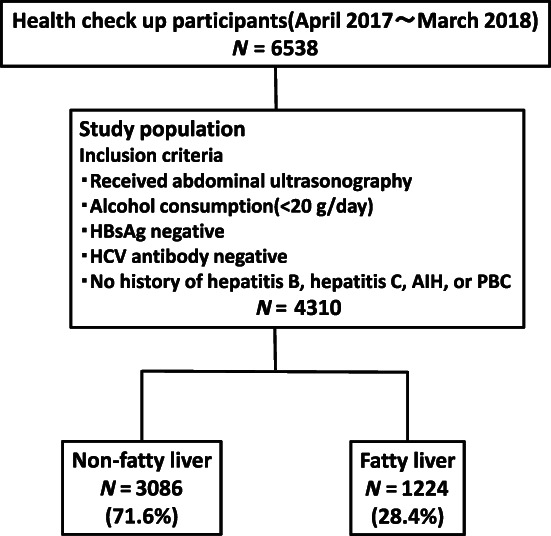
Study flow diagram. We studied 6538 participants. Participants were included if they fulfilled the following criteria: (i) they had undergone abdominal ultrasonography; (ii) they did not have alcoholic liver disease (alcohol intake of ≥20 g/day); (iii) they had no markers of hepatitis B virus (HBV) infection (hepatitis B surface antigen [HBsAg]) or hepatitis C virus (HCV) infection (anti‐HCV antibodies); and (iv) they had no history of HBV infection, HCV infection, autoimmune hepatitis (AIH), or primary biliary cholangitis (PBC). Finally, 4310 participants were enrolled. Of these, 1224 (28.4%) had nonalcoholic fatty liver disease (NAFLD).Study population *N* = 4310.

In total, 4310 participants were enrolled from April 2017 to March 2018. Among them, 1224 (28.4%) and 3086 (71.6%) participants did and did not have evidence of fatty liver on abdominal ultrasonography, respectively (Fig. 1).

### Prevalence of dyslipidemia, hypertension, and DM among participants undergoing health checkups

**Figure 2:**
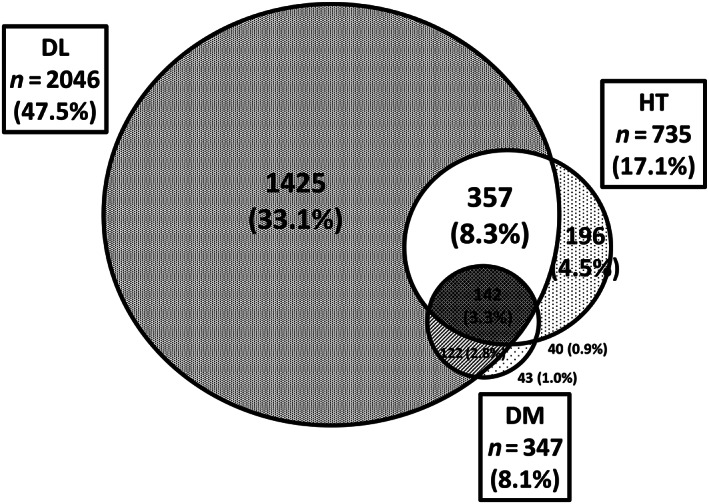
Prevalence of dyslipidemia (DL), hypertension (HT), and diabetes mellitus (DM) among participants undergoing health checkups. In total, 2046 (47.5%) of participants had DL, 735 (17.1%) had HT, and 347 (8.1%) had DM. A total of 1425 (33.1%) had only DL, 196 (4.5%) had only HT, and 43 (1.0%) had only DM. A total of 357 (8.3%) had DL and HT, 122 (2.8%) had DL and DM, 40 (0.9%) had HT and DM, and 142 (3.3%) had all three diseases. Many participants had only DL, but very few had only DM.

Of the 4310 participants who underwent abdominal ultrasound, 2046 (47.5%) had dyslipidemia, 735 (17.1%) had hypertension, and 347 (8.1%) had DM. A total of 1425 (33.1%) had only dyslipidemia (not including hypertension or DM), 196 (4.5%) had only hypertension (not including dyslipidemia or DM), and 43 (1.0%) had only DM (not including dyslipidemia or hypertension). Many participants had only dyslipidemia, but very few had only DM. A total of 357 (8.3%) participants had dyslipidemia and hypertension (not including DM), 122 (2.8%) had dyslipidemia and DM (not including hypertension), 40 (0.9%) had hypertension and DM (not including dyslipidemia), and 142 (3.3%) had dyslipidemia, hypertension, and DM. The most common complications were dyslipidemia and hypertension (Fig. 2).

### Prevalence of NAFLD in the dyslipidemia‐only, hypertension‐only, and DM‐only groups

**Figure 3:**
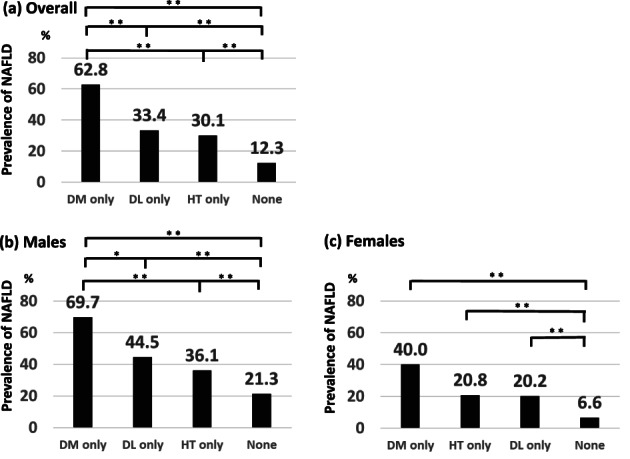
Prevalence of nonalcoholic fatty liver disease (NAFLD) in the dyslipidemia (DL)‐only, hypertension (HT)‐only, and diabetes mellitus (DM)‐only groups. Among all groups, the prevalence of NAFLD was highest in the DM‐only group. The prevalence of NAFLD in the DM‐only, DL‐only, and HT‐only groups were significantly different from that in participants with none of these three diseases (*P* < 0.001). DM only, DL(−)HT(−)DM(+); DL only, DL(+)HT(−)DM(−); HT only, DL(−)HT(+)DM(−); None, DL(−)HT(−)DM(−). **P < 0.001, *P < 0.005 (χ^2^‐test).

The prevalence of NAFLD was examined in the four groups of participants with only dyslipidemia (not including hypertension or DM), only hypertension (not including dyslipidemia or DM), only DM (not including dyslipidemia or hypertension), and none of these three diseases. Among the overall study cohort and among males, the prevalence of NAFLD decreased in the order of the DM‐only group, dyslipidemia‐only group, hypertension‐only group, and the group with none of these three diseases (Fig. 3a,b). Among females, the prevalence of NAFLD decreased in the order of DM‐only group, hypertension‐only group, and dyslipidemia‐only group, and the group with none of these three diseases (Fig. 3c). Among all groups, the prevalence of NAFLD was significantly higher in the DM‐only, dyslipidemia‐only, and hypertension‐only groups than in participants with none of these three diseases (*P* < 0.001). Among the overall study cohort, the prevalence of NAFLD was significantly higher in the DM‐only group than in the dyslipidemia‐only and hypertension‐only groups (*P* < 0.001) (Fig. 3a). Among males, the prevalence of NAFLD was significantly higher in the DM‐only group than in the dyslipidemia‐only group (*P* < 0.005) and the hypertension‐only group (*P* < 0.001) (Fig. 3b). The prevalence of NAFLD was higher in males than in females in all groups (Fig. 3).

### Comparison of the prevalence of non‐fatty liver and NAFLD in the dyslipidemia‐only, hypertension‐only, and DM‐only groups

**Figure 4:**
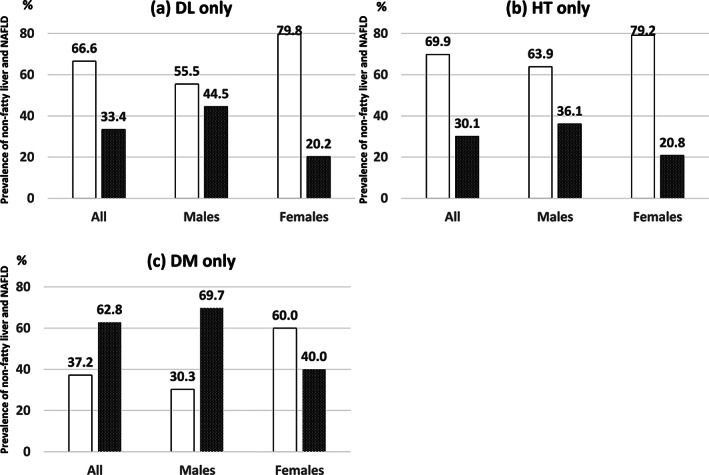
Comparison of the prevalence of non‐fatty liver and nonalcoholic fatty liver disease (NAFLD) in the dyslipidemia (DL)‐only, hypertension (HT)‐only, and diabetes mellitus (DM)‐only groups. The prevalence of non‐fatty liver was higher than that of NAFLD in the DL‐only and HT‐only groups. In the overall cohort and in males, the prevalence of NAFLD was higher than that of non‐fatty liver in the DM‐only group. The prevalence of NAFLD was higher in males than in females in all groups. 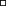, non‐fatty liver; 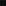, NAFLD.

Among the overall study cohort, in both males and females, the prevalence of NAFLD was lower than that of non‐fatty liver in the dyslipidemia‐only and hypertension‐only groups (Fig. 4a,b). In contrast, in the overall study cohort and in males, the prevalence of NAFLD was higher than that of non‐fatty liver in the DM‐only group (Fig. 4c). The prevalence of NAFLD was higher in males than in females in all groups (Fig. 4).

### Comparison of the prevalence of non‐fatty liver and NAFLD in the complication of dyslipidemia, hypertension, and DM groups

**Figure 5:**
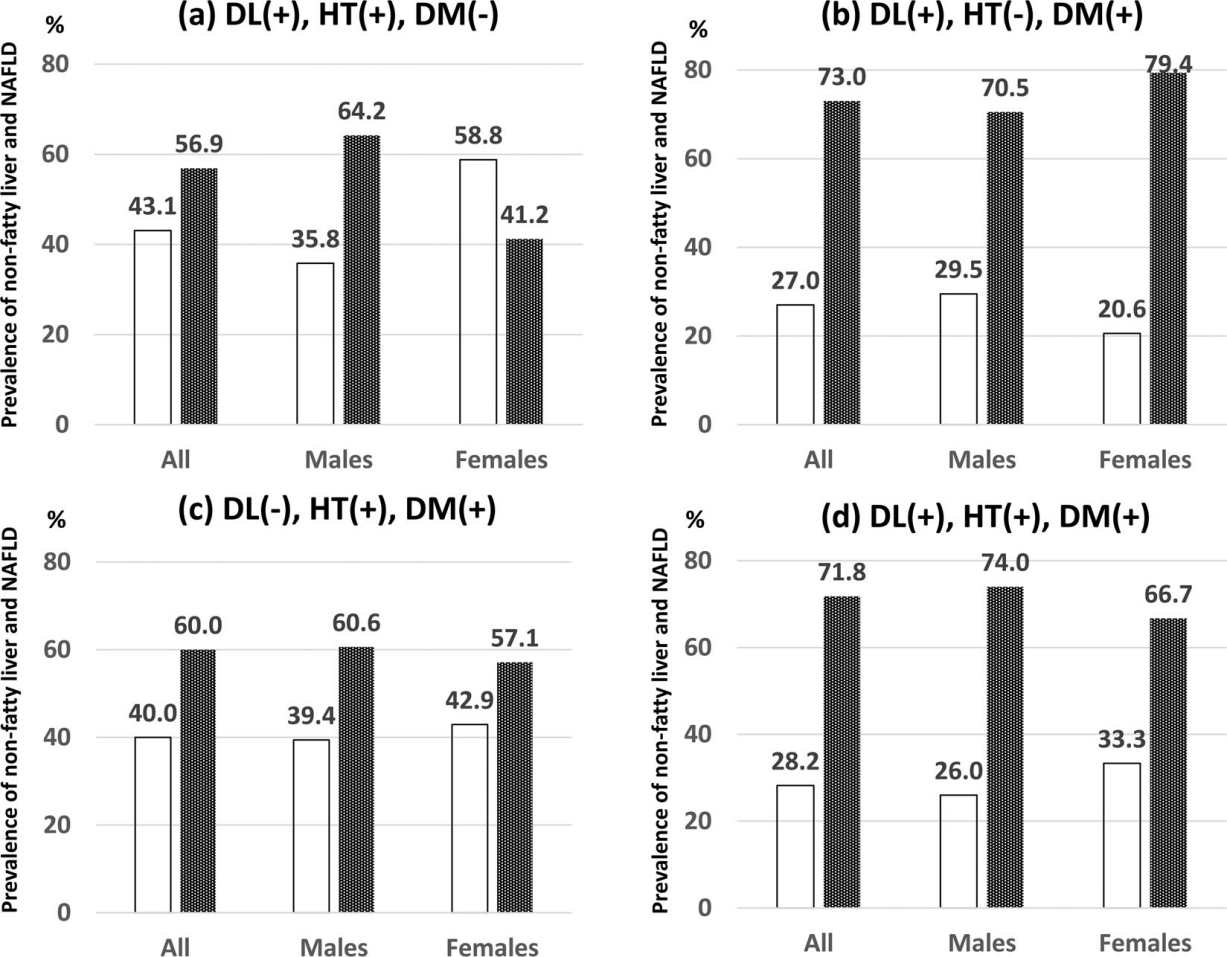
Comparison of the prevalence of non‐fatty liver and nonalcoholic fatty liver disease (NAFLD) in the complications of dyslipidemia (DL), hypertension (HT), and diabetes mellitus (DM). The prevalence of NAFLD was higher than that of non‐fatty liver in participants with complications of two or more factors of metabolic syndrome (DL, HT, and DM) except in females with DL and HT complications. 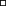, non‐fatty liver; 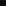, NAFLD.

In the overall study cohort and in males, the prevalence of NAFLD was higher than that of non‐fatty liver in the dyslipidemia and hypertension combined group (not including DM) (Fig. 5a). Among the overall study cohort, in both males and females, the prevalence of NAFLD was higher than that of non‐fatty liver in the dyslipidemia and DM combined group (not including hypertension) (Fig. 5b), in the hypertension and DM combined group (not including dyslipidemia) (Fig. 5c), and in the dyslipidemia, hypertension, and DM combined group (Fig. 5d). Notably, the prevalence of NAFLD was >70% in the dyslipidemia and DM combined group (not including hypertension) (Fig. 5b).

### Characteristics of non‐fatty liver and NAFLD

We found significant differences in all 16 items (age, gender, BMI, body fat percentage, waist circumference, systolic blood pressure, diastolic blood pressure, AST, ALT, LDL‐C, HDL‐C, triglycerides, fasting glucose, fasting insulin, HOMA‐IR, and HbA1c) between the non‐fatty liver group and the NAFLD group (*P* < 0.0001) (Table 1).

**Table 1:** Baseline characteristics between non‐fatty liver and nonalcoholic fatty liver disease (NAFLD)

	Non‐fatty liver *n* = 3086	NAFLD *n* = 1224	*P*‐value
Age (years)	49.6 ± 9.9	52.5 ± 8.6	<0.0001
Gender (M:F)	1275:1811	887:337	<0.0001
BMI (kg/m^2^)	21.8 ± 2.9	26.7 ± 4.1	<0.0001
Body fat percentage (%)	26.0 ± 7.0	29.8 ± 7.4	<0.0001
Waist circumference (cm)	77.5 ± 8.3	91.3 ± 10.3	<0.0001
sBP (mmHg)	111.8 ± 13.4	120.4 ± 12.9	<0.0001
dBP (mmHg)	67.4 ± 10.0	74.0 ± 9.8	<0.0001
AST (U/L)	20.0 ± 6.0	26.1 ± 13.2	<0.0001
ALT (U/L)	18.7 ± 9.2	35.9 ± 24.3	<0.0001
LDL‐C (mg/dL)	123.4 ± 30.0	134.3 ± 30.5	<0.0001
HDL‐C (mg/dL)	71.1 ± 17.1	55.2 ± 12.4	<0.0001
Triglycerides (mg/dl)	82.6 ± 42.0	142.9 ± 75.5	<0.0001
Fasting glucose (mg/dL)	98.6 ± 13.5	110.8 ± 21.2	<0.0001
Fasting insulin (μU/mL)	5.9 ± 3.2	11.9 ± 6.7	<0.0001
HOMA‐IR	1.5 ± 0.9	3.4 ± 2.3	<0.0001
HbA1c (%)	5.6 ± 0.4	6.0 ± 0.8	<0.0001

We found significant differences in all items. Values are expressed as mean ± standard deviation. A *P*‐value of <0.0001 was considered statistically significant by the Wilcoxon signed‐rank test.

ALT, alanine aminotransferase; AST, aspartate aminotransferase; dBP, diastolic blood pressure; sBP, systolic blood pressure.

### Identification of NAFLD‐related factors

Among the above 16 items, those that showed significant differences in the multivariate analysis were age, gender, body fat percentage, waist circumference, AST, ALT, HDL‐C, triglycerides, fasting insulin, and HbA1c (*P* < 0.005). In particular, gender (male/female) (odds ratio [OR], 2.62; 95% confidence interval [CI]: 1.39–4.93) and HbA1c (OR, 2.11; 95% CI: 1.57–2.82) were considered to be the independent factors most strongly associated with NAFLD (Table 2).

**Table 2:** Multivariate analysis of risk factors associated with nonalcoholic fatty liver disease (NAFLD)

	Multivariate analysis		
	OR	95% CI	*P*‐value
Age	1.02	1.01–1.03	0.0014
Gender (male/female)	2.62	1.39–4.93	0.0028
BMI	1.01	0.93–1.09	0.8385
Body fat percentage	1.08	1.03–1.13	0.0013
Waist circumference	1.06	1.03–1.08	<0.0001
sBP	1.00	0.99–1.01	0.5283
dBP	1.02	1.00–1.03	0.0163
AST	0.96	0.94–0.98	0.0002
ALT	1.07	1.05–1.08	<0.0001
LDL‐C	1.00	1.00–1.00	0.3239
HDL‐C	0.98	0.97–0.99	<0.0001
Triglycerides	1.01	1.00–1.01	<0.0001
Fasting glucose	1.00	0.99–1.02	0.5994
Fasting insulin	1.29	1.11–1.50	0.0037
HOMA‐IR	0.60	0.35–1.04	0.1018
HbA1c	2.11	1.57–2.82	<0.0001

Significant differences were found for age, gender, body fat percentage, waist circumference, aspartate aminotransferase (AST), alanine aminotransferase (ALT), HDL‐C, triglycerides, fasting insulin, and HbA1c (*P* < 0.005). Gender (male/female) (odds ratio [OR], 2.62; 95% confidence interval [CI]: 1.39–4.93) and HbA1c (OR, 2.11; 95% CI: 1.57–2.82) were considered the independent factors most strongly associated with NAFLD.

dBP, diastolic blood pressure; sBP, systolic blood pressure.

### Cutoff value for HbA1c

**Figure 6:**
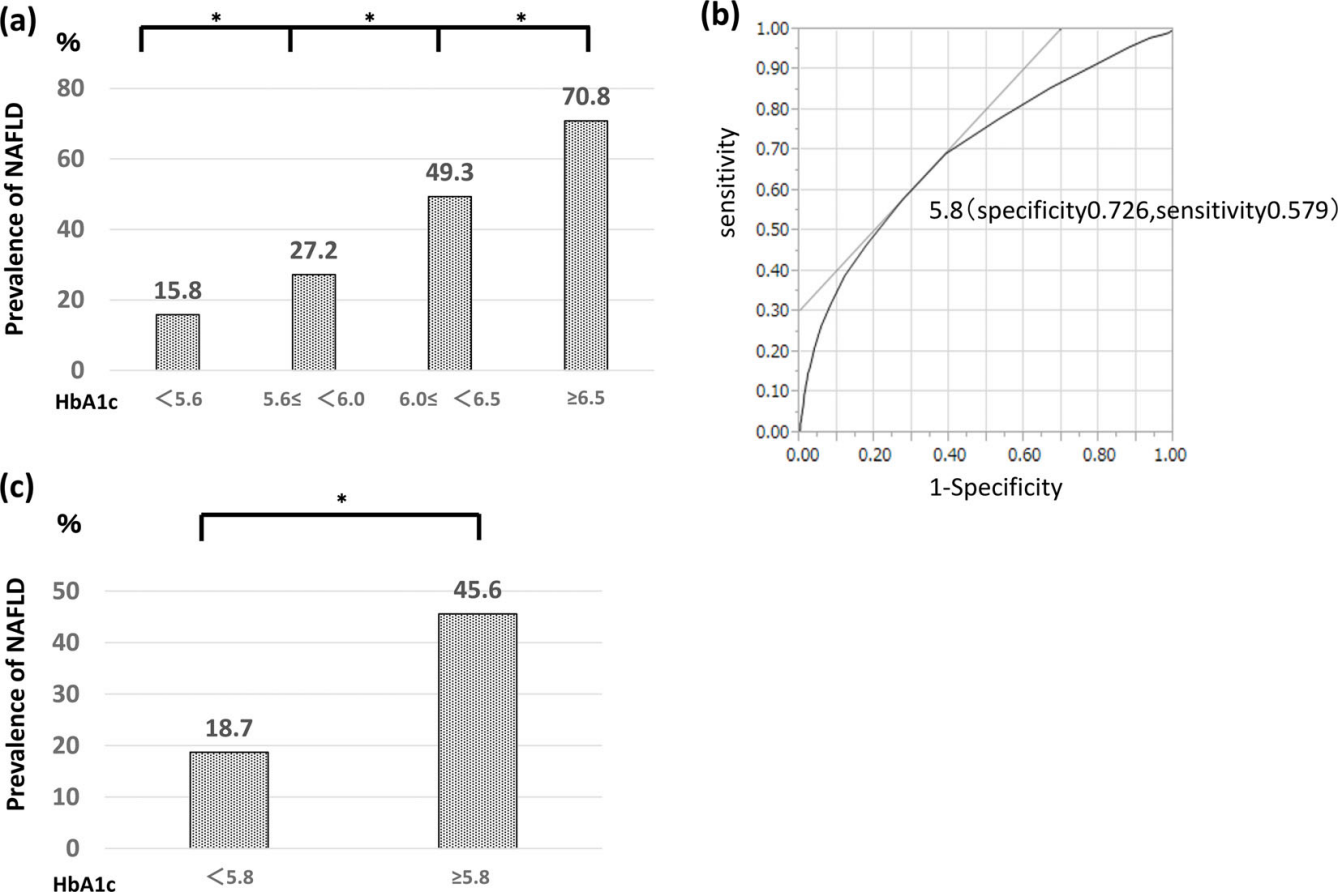
Cutoff value for HbA1c. The prevalence of nonalcoholic fatty liver disease (NAFLD) decreased in the order of an HbA1c level of ≥6.5% (70.8%), 6.0–6.5% (49.3%), 5.6–6.0% (27.2%), and <5.6% (15.8%). There was a significant difference between the groups (*P* < 0.0001). The HbA1c cutoff value was 5.8% (sensitivity, 57.9%; specificity, 72.6%; area under the curve, 0.70) by receiver operating characteristic curve. The prevalence of NAFLD was significantly higher in participants with an HbA1c level of ≥5.8% (45.6%) than <5.8% (18.7%) (*P* < 0.0001). HbA1c, hemoglobin A1c. *P < 0.0001 (χ^2^‐test).

The prevalence of NAFLD decreased in the order of participants with an HbA1c level of ≥6.5% (70.8%), 6.0–6.5% (49.3%), 5.6–6.0% (27.2%), and < 5.6% (15.8%), with significant differences (*P* < 0.0001) (Fig. 6a). The cutoff value for HbA1c by ROC analysis was 5.8% (sensitivity, 57.9%; specificity, 72.6%; area under the curve, 0.70) (Fig. 6b). The prevalence of NAFLD was significantly higher in participants with an HbA1c level of ≥5.8% (45.6%) than <5.8% (18.7%) (*P* < 0.0001) (Fig. 6c).

## Discussion

This study showed that the prevalence of NAFLD was highest in the DM‐only group among each factor of metabolic syndrome, although the prevalence of DM‐only was the lowest among all participants (Figs. 2 and 3). In addition, the prevalence of NAFLD was higher than that of non‐fatty liver in the DM‐only group, whereas the prevalence of NAFLD was lower than that of non‐fatty liver in the dyslipidemia‐only and hypertension‐only groups (Fig. 4). The prevalence of non‐fatty liver was higher than that of NAFLD among females in the DM‐only group, but this may have been due to the smaller number of females (*n* = 10) in the DM‐only group.

Previous studies have shown that 75% of patients with type 2 DM have NAFLD^14^ and that patients with NAFLD have a 2.22‐fold higher risk of developing DM compared with patients without NAFLD.^15^ In Japan, Jimba et al.^16^ investigated the association between impaired glucose tolerance and NAFLD in 1950 participants who underwent health checkups. The authors reported that 27% of participants who had a fasting glucose concentration within the reference level, 43% of those with impaired glucose tolerance, and 62% of those with DM had NAFLD and that the degree of impaired glucose tolerance was associated with the prevalence of NAFLD.^16^ Control of DM is also important in patients with NAFLD; reports have indicated that DM control is associated with liver fibrosis, that strict glycemic control prevents histological progression,^17^ and that DM doubles the risk of liver carcinogenesis.^18^

We now discuss the association between NAFLD and dyslipidemia. In one study, dyslipidemia was a complication in 65.7% of 1365 patients with NAFLD in Japan,^19^ and dyslipidemia has been shown to be a strong risk factor for the development of NAFLD.^8^ Increased lipolysis, increased uptake of free fatty acids by the liver, increased production of very‐low‐density lipoproteins, reduced free fatty acid oxidation, and decreased triglyceride efflux cause lipid accumulation in the liver, and these changes are associated with inflammation, oxidative stress, and the production of adipokines and cytokines.^20^ However, DM is reportedly an independent predictor of all‐cause and cardiovascular mortality in patients with NAFLD, but the use of statins and other lipid‐lowering drugs is not associated with reduced all‐cause or cardiovascular mortality.^21^ It has been suggested that pathogenic abnormalities that increase mortality in patients with NAFLD may not be targeted by treatment of lipid disorders alone. In our multivariate analysis, the OR was higher for HbA1c than for HDL‐C and triglycerides (Table 2). These data collectively indicate that the association with NAFLD is stronger for DM than for dyslipidemia.

Next, we will discuss the association of NAFLD with hypertension. In the above‐mentioned study, hypertension was a complication in 30.2% of 1365 patients with NAFLD in Japan.^19^ Hypertension and NAFLD are reportedly associated independently of other components of the metabolic syndrome. Hypertension and NAFLD contribute to each other's progression, and the mechanisms of this interaction are insulin resistance, activation of the renin–angiotensin–aldosterone system and the sympathetic nervous system, and arterial stiffness. Among these, insulin resistance is the major linkage mechanism.^22^ Conversely, hypertension is not associated with the development of NAFLD to the same extent as other components of metabolic syndrome or genetic predisposition, and whether hypertension is a risk factor for the development of NAFLD remains unclear.^8^ Hypertension is strongly associated with progression of fibrosis in patients with NAFLD,^23^ and antihypertensive treatment with angiotensin receptor blockers for NAFLD complicated by hypertension has been shown to reduce insulin resistance and progression of fibrosis.^22^ One of the mechanisms underlying the association between hypertension and NAFLD is insulin resistance. Thus, although hypertension is an independent risk factor for NAFLD, the association between DM and NAFLD may be stronger.

The present study showed that the prevalence of NAFLD was higher in males than in females in the dyslipidemia‐only, hypertension‐only, and DM‐only groups (Figs. 3 and 4). The reported prevalence of NAFLD among participants undergoing health checkups in Japan is 41.0% in males and 17.7% in females.^4^ This difference has generally been explained by the influence of sex hormones. Females are protected from the development and progression of NAFLD/NASH by estrogen.^24^ Furthermore, the present study showed that the prevalence of NAFLD was considerably higher when two or more metabolic syndrome factors were combined (Fig. 5). The prevalence of NAFLD is markedly increased in such participants, especially in those with the combination of DM and dyslipidemia. With respect to the prevalence of NAFLD, the effect of two or more metabolic syndrome components may be more significant than the gender‐related difference.

The multivariate analysis in this study showed that the factors most strongly associated with NAFLD were gender (male) and HbA1c (Table 2). The prevalence of NAFLD gradually increased according to the increase in the HbA1c level (Fig. 6a). DM is diagnosed by a fasting blood glucose concentration of ≥126 mg/dL and an HbA1c of ≥6.5%. The 75‐g oral glucose tolerance test is recommended for patients with an HbA1c of 6.0–6.4%. In a Japanese study of 39 patients with NAFLD who underwent consecutive liver biopsies, the decrease in HbA1c was associated with improvement of liver fibrosis, and control of HbA1c was found to be the most important factor in improving liver fibrosis in patients with NAFLD.^17^ In a study of non‐diabetic subjects with HbA1c levels of ≤6.4%, NAFLD was strongly associated with HbA1c.^25^ In our study, the cutoff value for HbA1c, a factor that was strongly associated with NAFLD, was 5.8% (Fig. 6b). The prevalence of NAFLD was significantly higher in participants with HbA1c levels of ≥5.8% than <5.8% (Fig. 6c). Fatty liver was diagnosed by abdominal ultrasonography in the present study. In B‐mode abdominal ultrasonography, fatty liver can be diagnosed when fatty degeneration is found in >20% of hepatocytes with a sensitivity of 85% and specificity of 94%.^26^ However, the problem with abdominal ultrasonography is that it is a subjective method of evaluation and has low sensitivity in patients with fatty liver characterized by <20% fatty degeneration.^27^ Some patients may have mild NAFLD with less than 20% fatty degeneration of the liver and no evidence of fatty liver on abdominal ultrasonography. In addition, many potential NAFLD patients do not have the opportunity to undergo abdominal ultrasonography. Taken together, these data indicate that the actual prevalence of NAFLD might be much higher.

Therefore, it is important to detect NAFLD efficiently. In this study, we found that HbA1c is the most effective factor for the detection of NAFLD, and we recommend that patients with an HbA1c of 5.8% or higher undergo abdominal ultrasonography. This makes it possible to detect NAFLD. A single‐center study may have the advantage of providing more accurate assessment than a multicenter study because in multicenter studies, there are often differences in the criteria used for fatty liver among institutions. In conclusion, NAFLD was most strongly associated with DM among the metabolic syndrome components, and HbA1c was strongly associated with NAFLD. We recommend abdominal ultrasonography for NAFLD detection in patients with an HbA1c of 5.8% or higher in general practice and health checkups. This makes it possible to detect NAFLD.
